# WHY DON’T ELDERS ADOPT TWO-FACTOR AUTHENTICATION? BECAUSE THEY ARE EXCLUDED BY DESIGN

**DOI:** 10.1093/geroni/igz038.1186

**Published:** 2019-11-08

**Authors:** Sanchari Das, Joshua Streiff, Lesa L Huber, L Jean Camp

**Affiliations:** 1 Indiana University Bloomington, Bloomington, Indiana, United States; 2 Indiana University School of Public Health, Bloomington, Indiana, United States

## Abstract

Two-Factor Authentication (2FA) provides effective protection for online accounts by providing efficient and highly robust access control. Adoption and usability, however, remain challenges for such technologies. Most research on 2FA focuses on students or employees in the tech sector. For example, our research with student populations found that lack of adoption was primarily due to a lack user risk concern matched with confidence in their ‘strong’ password strategies. The situation for older adults (> 60 years) was quite different, as we discovered through detailed interviews and think-aloud protocols targeted at understanding the registration, after installation, and their (un)willingness to use 2FA. We focused our research on USB security hardware tokens; additionally, we asked about other 2FA strategies which the participants adopted (if any). Their lack of adoption of the devices stemmed from its shortfall of inclusive design. Most available security tokens which were compliant with tablets have very small form factors; nearly invisible in a purse, and easy to slip through a pocket. The larger security keys are device and browser (Google Chrome) dependent. The organizations which would be most invested in protecting older people -- retirement management funds, the Social Security Administration, Medicare, and banking institutions - reasonably do not adopt 2FA because of its lack of acceptability. Our negative result is that older adults are caught in a negative feedback loop where lack of adoption prevents availability, and vice versa. The positive result is that these concerns are straight-forward to overcome.

